# Thyroid follicular carcinoma-like renal tumor: A case report and literature review

**DOI:** 10.1097/MD.0000000000010815

**Published:** 2018-05-25

**Authors:** Yujie Zhang, Jing Yang, Mingfang Zhang, Zhaowei Meng, Wenjing Song, Long Yang, Liming Li, Dan Wang, Tao Shi

**Affiliations:** a Department of Pathology, Tianjin Medical University General Hospital; b Department of Pathology, Tianjin Medical University; c Department of Pathology, Tianjin First Center Hospital; d Department of Nuclear Medicine; e Department of Urinary Surgery, Tianjin Medical University General Hospital, Tianjin, China.

**Keywords:** histochemistry staining, immunohistochemistry, renal cell carcinoma, thyroid carcinoma, thyroid follicular carcinoma-like renal tumor

## Abstract

**Rationale:**

Thyroid follicular carcinoma-like renal tumor (TFCLRT) is a rare primary renal epithelial tumor that was first reported in 2006. We report a case diagnosed of TFCLRT by us to observe the pathological feature and analyze comparatively the clinical and pathologic characteristics with all cases of reviewed literatures.

**Patient concerns:**

A 54-year-old female patient had the urinary frequency with the symptom of right flank pain with a history of more than half a year of hypertension and received uterine fibroid resection 12 years ago. B-mode ultrasound examination and renal magnetic resonance showed a right renal sinus nodule.

**Diagnoses:**

Histopathology revealed thyroid follicle-like structures of different sizes, containing a colloid-like substance, while the periodic acid-Schiff (PAS) and diastase-resistant PAS staining confirmed that it was mucus protein. Immunohistochemical staining showed that it expresses the transcription factor PAX-8 but does not express the thyroid-specific antibodies TG and TTF-1.

**Interventions:**

The patient underwent a tumor enucleation of right kidney. No other treatment was conducted after surgery.

**Outcomes:**

No metastases to lymph nodes and other organs were found, and 9-months of follow-up did not reveal any tumor progression.

**Lessons:**

We should differentially diagnose the renal metastasis of thyroid follicular carcinoma or papillary carcinoma. Some related literatures reported that the tumour cells had significant heteromorphism, several of which metastasized to lymph nodes or distal organs. Its biological behavior need to be studied intensively by further expanding the number of cases.

## 1 Introduction

Thyroid follicular carcinoma-like renal tumor (TFCLRT) is a rare primary renal epithelial tumor that was first reported in 2006.^[1]^ Thus far, there have only been 39 cases reported around the globe, including 17 cases reported in China. The naming of TFCLRT varies; it was tentatively designated as a subtype of renal cell carcinoma in the 2016 version of the World Health Organization (WHO) kidney tumor classification and was named thyroid-like follicular renal cell carcinoma.^[2]^ Here, we report a case diagnosed with thyroid follicular carcinoma-like renal tumor, and we also conducted comparative analysis of the relevant literature from the clinical and pathological point of view to improve the understanding of this type of tumor.

## 2 Case presentation

### 2.1 Patient information

A 54-year-old female patient had urinary frequency with the symptom of flank pain 1 month before admission, with no gross hematuria. The patient had a previous history of hypertension for more than half a year; her highest blood pressure was 180/140 mm Hg. The patient was taking telmisartan, and her routine blood pressure was 150/95 mm Hg. The patient received a hysterectomy 12 years ago due to uterine fibroids. There was no history of thyroid or ectopic thyroid-related diseases. There was no history of renal diseases too.

### 2.2 Clinical findings

**Figure 1:**
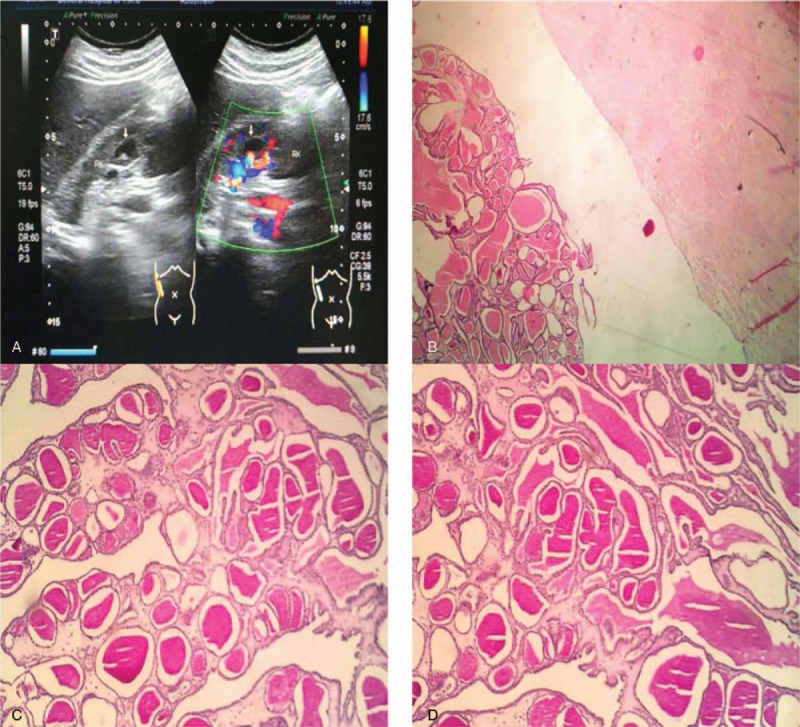
(A) B-mode ultrasound examination revealed a circle-like area without an echo signal located in the middle of the right kidney, which was approximately 1.9 × 1.6 cm in size with internal low-echo solid components and blood flow signal. (B) HE staining showed the tumor tissue is in the cavity of the dense fibrous tissue (×40). (C, D) PAS (C × 100) and D-PAS (D × 100) staining of the colloid-like substance were both positive, suggesting the presence of mucous protein. PAS = periodic acid-Schiff.

B-mode ultrasound examination revealed a circle-like area without an echo signal that was located in the middle of the right kidney in the vicinity of the intrarenal pelvis. It was approximately 1.9 × 1.6 cm in size and had internal low-echo solid components (Fig. 1A). Renal magnetic resonance showed a right renal sinus nodule, and positron emission tomography (PET) showed a slight hyperdense soft tissue nodule in the right renal sinus. Right kidney tumor enucleation was subsequently performed.

### 2.3 Diagnostic assessment

The surgical specimens were fixed with 4% neutral formaldehyde solution, followed by routine dehydration, paraffin embedding, sectioning into 3-μm-thick sections, hematoxylin and eosin (HE) staining, and light microscopic observation. Representative tissue blocks were also selected for immunohistochemical staining, periodic acid-Schiff (PAS) and diastase-resistant PAS (D-PAS) staining. The primary antibodies against PAX-8, cytokeratin 7 (CK7), EMA, cytokeratin 19 (CK19), CD10, vimentin, cytokeratin 20 (CK20), TTF-1, thyroglobulin (TG), and CD117 were from Beijing Zhongshan Golden Bridge Biotechnology Co., Ltd. A brownish color indicated positive staining. PAX-8 and TTF-1 were located in the nuclei; CK7, EMA, CK19, CD10, and CD117 were located in the cell membrane/cytoplasm; and vimentin and TG were located in the cytoplasm. PAS staining kit was from Guangzhou Wexis Biotech Co., Ltd. The specific staining procedures were performed according to the relevant manuals. All experimental processes were approved by the Ethical Committee of Tianjin Medical University General Hospital and the patient of the case report was contacted by telephone to obtain verbal informed consent.

**Figure 2:**
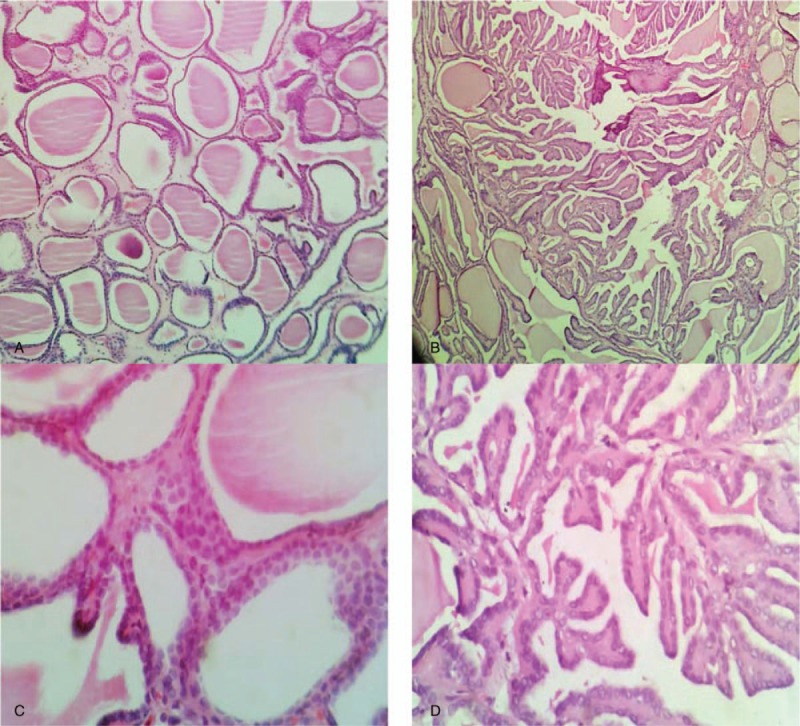
The tumor cells were arranged in two structures: a thyroid follicle-like structure with different sizes and full of a colloid-like substance (A × 100) and a papillary structure (B × 100). The two types of structures interweaved with each other, each accounting for approximately 50% of the cells. The cells were small cubic or columnar and the nuclei were round or oval (C × 400), while some were frosted glass-like, nuclear grooves could be occasionally found (D × 400).

Pathological features expressed that the tumor was nodular, with a partial thick capsule by general examination. It was approximately 2.5 × 2 × 1.5 cm in size, and the section appeared to be solid-cystic; the solid area was gray and carrion like. Microscopic manifestations were as follows. In the cavity of the dense fibrous tissue (Fig. 1B), tumor cells were roughly arranged in 2 structures (Fig. 2A and B): a thyroid follicle-like structure with different sizes and full of a colloid-like substance; and a papillary structure. The 2 types of structures interweaved with each other, each accounting for approximately 50% of the cells. The tumor cells were arranged in monolayers and were small cubic or columnar. Some tumor cells were flat due to oppression from the colloid-like substances. Cells had medium-rich cytoplasm and were predominantly amphiphilic or eosinophilic. Most of the nuclei were located in the basal part, were of medium size, and were round or oval (Fig. 2D), while some were frosted glass-like (mainly in the papillary area) (Fig. 2C); nuclear grooves could be occasionally found, and nuclear atypia was not significant (Fuhrman nuclear grade 1–2). A small amount of interstitial inflammatory cell infiltration could be observed, and local lesions contained aggregations of hemosiderin-containing cells.

**Figure 3:**
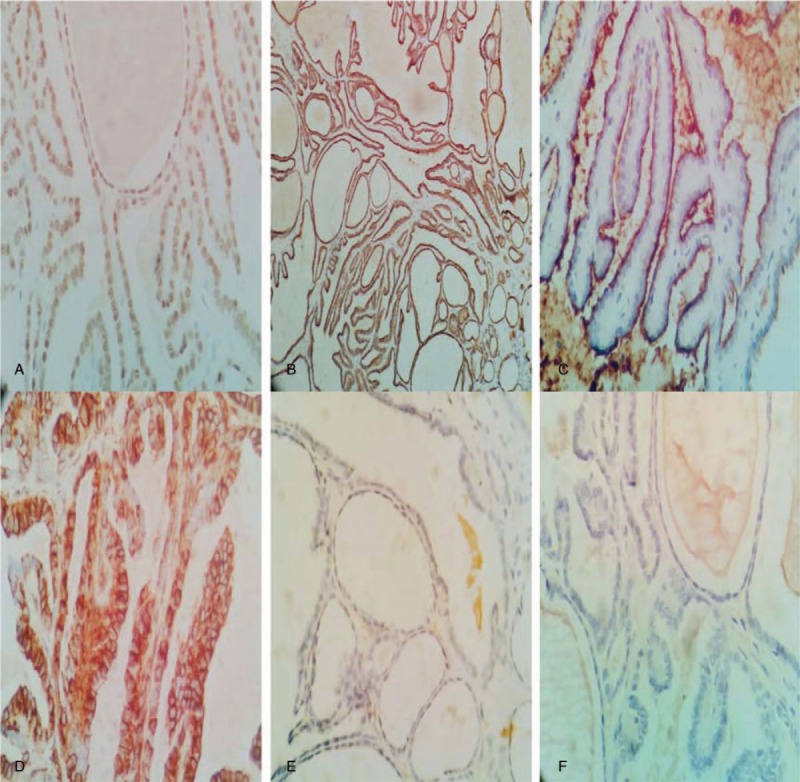
Immunohistochemical staining showed that PAX-8 (A × 400), CK7 (B × 100), CD10 (C × 400), and Vimentin (D × 400) were positive respectively located in the nuclei, the cytoplasm, the cellular cavity margin, the membrane and cytoplasm. Tg (E × 400) and TTF-1 (F × 400) were negative. PAS = periodic acid-Schiff.

Immunohistochemical staining (Fig. 3) and histochemical special staining (Fig. 1 C and D) results showed that tumor cell epithelial membrane antigen (EMA) (positive in the edge of cavities), cytokeratin-7 and 19, and PAX-8 were diffusively positive, CD10 (cavity margin positive) and vimentin were partially positive, and thyroid transcription factor (TTF-1) and thyroglobulin (TG) were both negative. The periodic acid-Schiff (PAS) and diastase-resistant PAS (D-PAS) staining of the colloid-like substance were both positive, supporting was mucous protein.

### 2.4 Follow-up and outcomes

No other treatment was conducted after surgery, no metastases to lymph nodes and other organs were found, and 9-months of follow-up did not reveal any tumor progression.

### 2.5 Literature review

Case reports in English were searched in the PubMed database using the keywords “thyroid” + “follicular” + “kidney”/“renal tumor”; 25 related reports were found. Case reports in Chinese were searched in the Wanfang database using the keywords “(thyroid)” + “(follicle)” + “(kidney)”; 15 related reports were found. We read and combined duplicated case reports and obtained a total of 39 cases, of which 17 cases were reported in China. These 39 case reports were reviewed and compared with this case (Table 1).

**Table 1:**
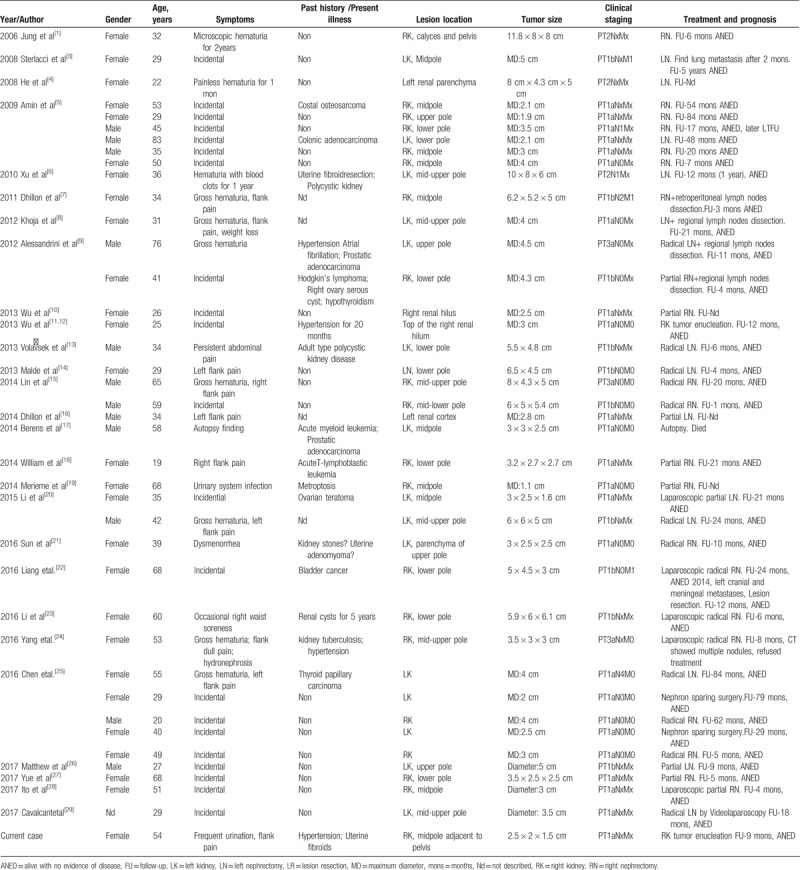
Clinical pathology conditions of 40 cases of TFCLRT.

## 3 Discussion

Based on naming conventions and biological behavior, the author who identified the first case of this type of tumor named it “thyroid follicular carcinoma-like tumor of kidney.”^[1]^ Subsequently, Sterlacci et al^[3]^ and He et al^[4]^ each reported one case and utilized similar names in their respective reports, with the keyword of “tumor”, suggesting that the biological behavior of this type of tumor is uncertain: The case reported by Sterlacci et al was found to have metastases in the left lower lobe of the lung 2 months after the surgery. In 2009, Amin et al^[5]^ reported 6 cases, of which one case was associated with renal portal lymph node metastasis (N1) and was named “primary thyroid-like follicular carcinoma of the kidney,” with the main word of “carcinoma” revealing its malignant biological behavior. Since then, case reports in China and abroad have all used the keyword “carcinoma,” of which some cases^[6,7,22,25]^ were characterized by renal portal lymph node metastasis (1/2), bilateral lung metastasis (4/4), retroperitoneal lymph node metastasis (2/17), and skull and meningeal metastasis. However, the lymph node metastasis rate was low (7/23), and distance metastases were mainly of metastases to the lung, skull, and meninges, suggesting that this tumor is capable of low malignancy. The 2016 version of the WHO kidney tumor classification^[2]^ tentatively designated this type of tumor as a subtype of renal cell carcinoma and named it thyroid-like follicular renal cell carcinoma. However, the pathology and biological behavior of our case are very benign. Given that all the reported patients have survived so far except for one who died of acute leukemia 16 and the prognosis is good, the naming of “thyroid follicular carcinoma-like renal tumor” was adopted in our final diagnostic report.

Clinical data^[1,3–29]^ showed more female patients than male patients were affected by this type of tumor (27/12 cases), and the age of onset was 19 to 83 years, with a mean age of 43 years and a median age of 39 years, of which 33 patients were in the range of 19 to 60 years, suggesting that the tumors often occur in young and middle-aged populations. Most cases were incidentally discovered (23 cases); some were associated with urinary system symptoms, mainly hematuria and flank pain (16 cases), and 1 case was discovered in autopsy. Our case was characterized by urinary frequency with flank pain symptoms, but no hematuria. Analysis of the disease history has found that patients with previous history mainly had urinary system diseases, reproductive system diseases, and hypertension, and there was no close association with thyroid tumors. All cases had a single lesion in one of the kidneys, with the incidence of tumors in the right kidney being slightly higher than that of tumors in the left kidney (22/18 cases). The tumors mainly occurred in the mid-pole of the kidney and the surrounding area; in this case, the tumor occurred in the mid-pole of the kidney adjacent to the renal sinus.

From the pathological point of view, the general morphological manifestations were solid cysts or solid nodules with fibrous capsules of different thickness, and the maximum diameter was in the range of 1.1 to 11.8 cm (with an average of 4.3 cm). There were 5 cases that showed invasion of the capsule, renal parenchyma, renal pelvis, renal capsule, and perirenal fat capsule and nerve tissue.^[4,9,15,24]^ The section surface was yellow and white to brown, the texture ranged from slightly tough to delicate, and some cases showed bleeding necrotic areas. Microscopic morphology revealed thyroid follicle-like structures of different sizes, containing an eosinophilic, amorphous, colloid-like substance, while special staining confirmed that the substance was mucus protein rather than thyroid gel and revealed that the substance could overflow the follicles. The morphology was similar to that of well-differentiated thyroid follicle cancer. Some cases had different proportions of papillary structure,^[3,9,13,18,20,25]^ similar to thyroid papillary carcinoma, and some were even reported to have complete papillary structure 8. Some cases with invasion or metastasis had visible solid patch-like, cord-like, or follicle-shaped structures with diverse morphologies,^[4–6,9,15,22]^ and the morphological heterogeneity of metastatic lesions also increased accordingly. The tumor interstitium was separated by fibrous connective tissues of different widths, and individual cases could have smooth muscle^[25]^ that could be associated with calcification or psammoma bodies, cholesterol crystallization, or hemorrhagic necrosis, while others could be associated with the infiltration of a large number of lymphocytes with the formation of germinal centers.^[5,10]^ Cells were in monolayers and were cubic or short columnar. Some cells could appear flat due to the oppression of the inclusion content. Cells mainly had eosinophilic cytoplasm, and some showed transparent cytoplasm. The nuclei were round or oval, with vague nucleoli, and some cases showed nuclear grooves, frosted glass-like nuclei, or nuclear pseudo-inclusion bodies. Nuclear division phases were rare; most of the nuclei were grade 2, and some cases had significant nuclear atypia.^[1,6,13,15,22,25]^

Immunohistochemical staining studies in the literatures showed that this tumor consistently expressed the transcription factor PAX-8 but did not express the thyroid-specific antibodies TG and TTF-1. Almost all cases had positive expression of CK (pan), vimentin, EMA, CK7, and CK19 and had low expression of Ki-67. Some cases had various degrees of positive CD10 and RCC expression, but there was only 1 case with positive CD117 expression.^[23]^ Individual cases had weak positive expression of the neuroendocrine markers (Syn, CgA, CD56, and NSE).^[4]^ We believe that for diagnosis of the tumor, PAX-8, TTF-1, and TG immunohistochemistry could be applied on the basis of morphological analysis, which could be further combined with analyses of CD10, EMA, vimentin, CK7, and CK19 expression levels.

Several similar diseases need to be differentially diagnosed, as follows. Renal metastasis of thyroid follicular carcinoma or papillary carcinoma is very rare, with less than 20 cases reported in the literature,^[30–32]^ and requires comprehensive identification based on the combination of disease history, thyroid-related examination, and immunohistochemistry. Renal primary clear cell, papillary, or cystic renal cell carcinoma is characterized by all 3 tumors showing no follicle-like structures and eosinophilic colloid-like substances. Diagnoses can be made based on the clinical data of various subtypes of renal cell carcinoma, histology, and related immunohistochemistry studies. Ovarian goiter in female patients can metastasize, though at a rate of only 5%, and mainly to the liver and peritoneum,^[33]^ with no report on metastasis to the kidney thus far. However, the possibility of metastasis to the kidney needs to be excluded via imaging examination excluding ovarian space-occupying lesions combined with immunohistochemistry. Renal thyroidization often occurs secondary to chronic pyelonephritis, obstructive urinary tract disease, and advanced nephropathy and often consists of non-neoplastic lesions involving both sides of the kidney, without the formation of localized lesions.

Regarding treatment and prognosis, surgical resection is the main treatment method for TFCLRT. According to the literature,^[1,3–29]^ most tumors with diameters of more than 4 cm or associated with invasive growth or distant metastasis are treated mainly with radical nephrectomy, combined with the corresponding regional lymph node dissection; tumors with diameters of < 6 cm, a complete capsule, and no metastasis are mainly treated with partial nephrectomy or tumor enucleation. The available data suggest that TFCLRT is a type of tumor with a low degree of malignancy, a low recurrence rate (1 case of suspected recurrence^[24]^), and a low rate of metastasis (5/40 cases) and has minimal progression after surgery.

At present, the understanding of TFCLRT is still very limited. We need to further expand the number of cases and should perform in-depth investigations with longer periods of follow-up to gain more in-depth understanding.

## Author contributions

**Data curation:** Zhaowei Meng, Wenjing Song, Liming Li.

**Formal analysis:** Zhaowei Meng.

**Resources:** Wenjing Song, Long Yang, Tao Shi, Dan Wang.

**Writing – original draft:** Yujie Zhang.

**Writing – review & editing:** Jing Yang, Mingfang Zhang.
